# Education in Computational Biology Today and Tomorrow

**DOI:** 10.1371/journal.pcbi.1003391

**Published:** 2013-12-12

**Authors:** Joanne A. Fox, B. F. Francis Ouellette

**Affiliations:** 1 Michael Smith Laboratories, University of British Columbia, Vancouver, British Columbia, Canada; 2 Department of Microbiology & Immunology, University of British Columbia, Vancouver, British Columbia, Canada; 3 Ontario Institute for Cancer Research, Toronto, Canada; 4 Department of Cell and Systems Biology, University of Toronto, Toronto, Canada

The etymology of the word “education” in Wikipedia is enlightening: “a rearing” and “I lead forth” (http://en.wikipedia.org/wiki/Education#Etymology). Computational biology educators are leading and raising the next generation of scientists and, in doing so, are in need of new tools, methods, and approaches. The need for education in science, and in computational biology in particular, is greater than ever. Large datasets, -omics technologies, and overlapping domains permeate many of the big research questions of our day. *PLOS Computational Biology* originally created the Education section to highlight the importance of education in the field [1]. Thus, it was a great honor when Fran Lewitter, Education Editor for the past eight years, along with Philip E. Bourne and Ruth Nussinov, contacted us to work as editors of the *PLOS Computational Biology* Education section. In our minds, educational initiatives in computational biology and bioinformatics serve two important goals: to communicate digital biology with each other, and to educate others on how best to do this. These are themes we practice as educators in our university teaching, in our involvement with the bioinfomatics.ca workshops series, and in our outreach efforts. We are very excited to continue Fran's great vision as we continue her work with the *PLOS Computational Biology* staff.

Examples of tutorials, specialized workshops, and outreach programs that bridge the knowledge gap created by this fast pace of research have been previously highlighted in this collection. There have been several types of articles, but two stand out. Firstly, there are tutorials about a specific biological problem requiring a specific approach, tools, and databases. For example, ”Practical Strategies for Discovering Regulatory DNA Sequence Motifs„ by MacIsaak and Fraenkel [2]. Tutorial articles provide theoretical context, as well as the type of questions and how to answer them. The other type of article we frequently find in the Education collection are “primers” or “quick guides.” For example, Eglen's “A Quick Guide to Teaching R Programming to Computational Biology Students” [3] or Bassi's “A Primer on Python for Life Science Researchers” [4]. Both of these examples from the Education collection address an important niche within the community. The “Quick Guide” series provides a more generic introduction to an approach in computational biology that can be applied across multiple domains. All of these types of articles will continue to be well-supported and encouraged in the Education collection. Many other articles have also been well-received, and seem to address gaps in the education material. We want to revisit older collection papers and identify where methods and technologies have evolved to a point where new methods are now in use, and invite previous or new authors to contribute.

These initiatives help to extend computational biology beyond the domain of specialized laboratories. Researchers, at all levels, need to keep themselves up-to-date with the quickly changing world of computational biology, and trainees need programs where bioinformatics skills are embedded so they can have comprehensive training. New bioinformatics workflows can be adopted more widely if education efforts keep pace. As previously pointed out [5], starting early is also very important. There is still room for programs that capture the excitement and enthusiasm of secondary school students and convey the potential of computational biology to the public. We welcome additions to the *PLOS Computational Biology* “Bioinformatics: Starting Early” collection (www.ploscollections.org/cbstartingearly).

We would like to involve the community in this endeavor. With this editorial, we are calling out to educators and researchers who have experience in teaching, specifically, those keen to raise the expectations and the inquisitiveness of the next generation of biologists. The Education collection will continue to publish leading edge education materials in the form of tutorials that can be used in a “classroom” setting (whatever that may mean nowadays: stated more generically, “the places where people learn”). We will continue to encourage articles set in the context of addressing a particular biological question and, as mentioned above, we welcome new “primers” and “quick guides.” We will also be inviting tutorials from the various computational meetings. A new category of papers that is in the pipeline for the Education collection is the “Quick Tips” format, the first of which was just published [6]. The “Quick Tips” articles address specific tools or databases that are in wide use in the community.

We also hope, and plan, to incorporate new thinking and perspectives in the greater field of education of computational biology and bioinformatics. For example, articles that highlight the use of new tools such as those used in cloud computing or methods for using third and fourth generation sequencing technologies are encouraged. We would also like to see articles that incorporate best practices in teaching, including the use of new media, flexible online teaching tools, and the use and re-use of large well-defined data sets that are computed on in classes, courses, and programs. We encourage articles that highlight new types of training initiatives, the use of workflows to help students in the path to reproducibility in science, and open course materials (open lecture notes and open course notes and datasets for exercises) that reach more learners.

In the end, the Education section belongs to the community and thus comes with responsibilities. We need to identify the gaps and the material with which we want to educate ourselves; we need to recognize and encourage great teachers and writers to communicate openly about what works best with the specific methods. We invite you to contact us via ploscompbiol@plos.org with your ideas for the kind of articles you would like to see in the *PLOS Computational Biology* Education section. We hope to see you in the classroom soon, where we learn together.

**About The Authors**

**Joanne A. Fox** (@joannealisonfox on Twitter) has a PhD in Genetics from the University of British Columbia (UBC). As a faculty member at the Michael Smith Laboratories and in the Department of Microbiology and Immunology at UBC, she is involved in a range of education and outreach initiatives at the undergraduate and secondary school levels, and teaches a variety of courses. She is a former instructor and current review committee member of the Canadian Bioinformatics.ca Workshops.

**B.F. Francis Ouellette** (@bffo on Twitter) did his graduate studies in Developmental Biology and is now an Associate Professor in Cell and Systems Biology at the University of Toronto, as well as a senior scientist and Associate Director of Informatics and Biocomputing at the Ontario Institute for Cancer Research. He was one of the founders and is still the scientific director and an instructor for the Canadian Bioinformatics.ca Workshops.

The authors have worked together in the past, and have known each other for more than 15 years.
